# Retraction of “Effect of C-phycocyanin on HDAC3 and miRNA-335 in Alzheimer’s disease”

**DOI:** 10.1515/tnsci-2022-0808

**Published:** 2024-05-31

**Authors:** Zhengyu Li, Li Gan, Si Yan, Yufang Yan, Wei Huang

**Affiliations:** Department of Neurology, The Second Affiliated Hospital of Nanchang University, Nanchang, Jiangxi, 330006, China

The publisher would like to inform that the article “Effect of C-phycocyanin on HDAC3 and miRNA-335 in Alzheimer’s disease” (https://doi.org/10.1515/tnsci-2020-0101) has been retracted at the request of the Editors.

The retraction is due to the discovery that Figure 3E is identical to Figure 6D in the previously published paper by Chen P, Chen F, and Zhou B titled “Antioxidative, anti-inflammatory and anti-apoptotic effects of ellagic acid in liver and brain of rats treated by d-galactose,” published in Sci Rep, 2018 Jan;8(1):1465.

This overlap compromises the originality and integrity of the results, and the final conclusions of the article could not be confirmed.

